# Bridge2AI recommendations for AI-ready genomic data

**DOI:** 10.1093/bioadv/vbag305

**Published:** 2026-10-25

**Authors:** Matthew Cannon, Wesley Goar, In-Hee Lee, James Stevenson, Amy Heiser, Nathan C Sheffield, James Eddy, Monica Munoz-Torres, Sek Won Kong, Alex H Wagner

**Affiliations:** Institute for Genomic Medicine (IGM), Nationwide Children’s Hospital, Columbus, OH, USA; Institute for Genomic Medicine (IGM), Nationwide Children’s Hospital, Columbus, OH, USA; Computational Health Informatics Program (CHIP), Boston Children’s Hospital, Boston, MA, USA; Institute for Genomic Medicine (IGM), Nationwide Children’s Hospital, Columbus, OH, USA; Sage Bionetworks, Seattle, WA, USA; Department of Genome Sciences, University of Virginia, Charlottesville, VA, USA; Avantiqor, Washington, DC, USA; Department of Biomedical Informatics, University of Colorado Anschutz Medical Campus, Aurora, CO, USA; Computational Health Informatics Program (CHIP), Boston Children’s Hospital, Boston, MA, USA; Department of Pediatrics, Harvard Medical School, Boston, MA, USA; Institute for Genomic Medicine (IGM), Nationwide Children’s Hospital, Columbus, OH, USA; Department of Pediatrics, The Ohio State University College of Medicine, Columbus, OH, USA

## Abstract

Rapid advancements in technology have led to an increased use of artificial intelligence (AI) technologies in medicine and bioinformatics research. In anticipation of this, the National Institutes of Health (NIH) assembled the Bridge to Artificial Intelligence (Bridge2AI) consortium to coordinate development of “AI-ready” datasets that can be leveraged by AI models to address grand challenges in human health and disease. The widespread availability of genome sequencing technologies for biomedical research presents a key data type for informing AI models, necessitating that genomics data sets are “AI-ready.” To this end, the Genomic Information Standards Team (GIST) of the Bridge2AI Standards Working Group has documented a set of recommendations for maintaining AI-ready genomics datasets. In this report, we describe recommendations for the collection, storage, identification, and proper use of genomics datasets to enable them to be considered “AI-ready” and thus drive new insights in medicine through AI and machine learning applications.

## 1 Introduction

### 1.1. Motivation

State-of-the-art artificial intelligence (AI) technologies are now being applied to biology and bioinformatics research problems across the world to great effect. Despite their widespread adoption and applications for large biological (and clinical) datasets, the definition of what an AI-ready dataset looks like has only recently been discussed. Led by the U.S. National Institutes of Health (NIH) Bridge to Artificial Intelligence (Bridge2AI) consortium, AI-ready datasets are those that are FAIR, fully reliable, robustly defined, and computationally accessible (Caufield *et al*. 2025, Clark *et al.* 2026). Using these assessments as a guide, Bridge2AI data generation projects such as “Cell Maps for Artificial Intelligence” (CM4AI) have created robust, reusable, and explainable biomedical datasets that can be used with AI applications to improve our understanding of human health and our ability to treat human disease (Clark *et al*. 2024b). Given the power of AI technologies for generating new insights in medicine, our ability to prepare datasets and provide provenance for these applications will be essential for driving new discoveries in existing medical fields.

Advances in sequencing technologies and bioinformatics have led to an exponential increase in the amount of genomic sequencing data being generated for biomedical studies (Brittain *et al*. 2017, Mani *et al*. 2025). Accompanying this growth is a commensurate opportunity for enabling these data (and associated downstream data such as gene expression quantification and sequence variant calls) for use in AI applications. However, the diversity of sequencing methods varies substantially, creating barriers to automated reuse of these data to drive meaningful breakthroughs and insights via AI applications. Explainability and reproducibility at all stages of data collection and model development is therefore essential to support trust in discoveries generated through AI affiliated methodologies (Al Manir *et al*. 2021, Levinson *et al*. 2022).

To guide development of the Bridge2AI data generation projects and broader community efforts to construct AI-ready genomic datasets, the Bridge2AI Genomic Information Standards Team (GIST) has developed the following summary of data and metadata recommendations for genomic sequencing experiments to support AI-driven biomedical discovery. These recommendations build upon the principles of AI-readiness for biomedical data (Clark *et al.* 2026), and broadly describe the sufficient metadata necessary for robust and explainable datasets that are suitable for use in downstream AI applications. While many of these recommendations are consistent with FAIR data principles, we place additional emphasis on metadata that enable automated interpretation and reproducible analysis. Metadata that might be optional for human interpretation often become essential for AI systems where experimental context cannot be easily inferred. Further, while modern AI methods can often extract information from free text, we recommend structured metadata using established schemas and controlled vocabularies whenever possible to maximize machine readability. Accordingly, our recommendations emphasize metadata that minimize ambiguity, facilitate automation across datasets, and support reproducibility of AI-derived results.

In this work, we cover the collection, storage, identification, and use of genomic sequencing data to meet these criteria. The remit of these recommendations is genomic DNA sequencing data (i.e. “sequencing reads” and downstream artifacts), with an emphasis on short-read sequencing workflows and small variant analyses that currently represent the predominant use cases in human genomics. While many of the metadata and provenance recommendations described here are broadly applicable to other genomics technologies (e.g. long-read sequencing, single-cell genomics, epigenomics, structural variant detection), these modalities introduce additional considerations that are still evolving and are beyond the scope of this Perspective. Additionally, while consent and clinical phenotype metadata should be included, we will not discuss these to keep our recommendations modular and focused on genomic data and its pre- and post-processing.

## 2 Main text

Central to AI-readiness is the need for the data to contain the necessary metadata for automated and manual interpretation, evaluation, and validation. The metadata we describe below is what must or should be included to control for covariates during the processing of genomic sequence data. Definitions for genomics metadata terminology and file formats throughout this report have been assembled from established ontologies and are provided in Table S1, available at supplementary material  *Bioinformatics Advances* online (Ong *et al*. 2017, van der Velde *et al*. 2022, McLaughlin *et al*. 2025). A more detailed list of possible metadata to ensure findable, accessible, interoperable, and reusable (FAIR) genomes can be found at FAIR Genomes, EDAM, MIxS GSC, Sequence Ontology, EFO, and NCIT, and should be used when possible (Eilbeck *et al*. 2005, Field *et al*. 2008, Malone *et al*. 2010, Black *et al*. 2022, van der Velde *et al*. 2022). While no single ontology is recommended, metadata should be represented using established community standards and linked through cross-ontology mappings to facilitate interoperability across datasets. All datasets must include a metadata file describing (1) what each file in the dataset contains, (2) the date the dataset was generated, (3) data processing methods used, (4) site where the file was processed, and (5) an email address for the study’s contact individual. The description of file contents should describe each data field with corresponding data types using community-accepted metadata standards while ensuring privacy. To balance reproducibility with participant privacy, metadata should be reported at the highest level of granularity permitted by participant consent and institutional governance (i.e. with the greatest level of detail that can be shared responsibly).

The recommendation levels (“Must,” “Should,” “May”) were developed through expert consensus within the Bridge2AI Genomic Information Standards Team. These requirement levels follow the conventions described in RFC 2119, where “Must” denotes metadata considered essential for interpretation and reuse of genomic datasets, “Should” denotes metadata that improves AI-readiness but may not be available in every context, and “May” denotes optional metadata that can provide additional value when available. Assignment of these requirement levels in the context of AI-readiness considered the necessity of a metadata element for reproducibility, interoperability, and its necessity for enabling interpretation across diverse genomic sequencing studies. “Must” designates information necessary to understand what was generated, how it was produced, or what can be reused. “Should” designates information that substantially improves downstream interpretation or AI-enabled analysis but may not be universally applicable or available across studies. “May” designates optional information that can provide additional context when available. Thus, a field may be highly informative for a particular use case while remaining a “Should” when it is not required to interpret or reuse every genomic sequencing dataset. Conversely, fields that are essential only for certain study designs or specimen types are designated “Must if applicable.” In the following sections, we provide recommendations for relevant metadata fields to include, as well as references to community-accepted standards for each data type and example values where applicable.

### 2.1 Description of supporting metadata for sample origin and preparation

Characteristics of the originating sample used for genomic sequencing that describe the source, sample quality and provenance (summarized in Table 1) are necessary for informing downstream analyses. Sample preservation and storage conditions must be included and should describe in detail how the genomic sample was stored and maintained, e.g. if samples were fresh, flash frozen, or stored in formalin-fixed paraffin-embedded blocks. These metadata inform downstream AI and human agents on processing limitations and potential biases inherent in the source sample; for example, formalin-fixed tissue is more degraded than other storage methods, such as flash frozen samples (Guyard *et al*. 2017), adversely affecting read quality. We also recommend including dates and physical locations of sample collection and sample preparation, the sampling protocol, biospecimen type (e.g. tissue or cell culture), and where applicable, clinical diagnosis, and pathological state (e.g. normal or tumor), all of which should be included within this subset of metadata (Table 1). If the sample is derived from a human-derived specimen, the metadata should include sex assigned at birth, genetic ancestry, and clinical phenotypes. These metadata should be captured using precise and well-defined terminology. Genetic ancestry should be distinguished from self-reported race or ethnicity, while sex assigned at birth, gender identity, and genotypic sex represent distinct concepts that should not be used interchangeably. Where metadata are inferred rather than directly reported, the method of inference should be documented.

**Table 1:** ** **Metadata recommendations to describe sample provenance and sequencing acquisition.

Requirement level	Name	Standards used	Sample from
**Must**	Storage Conditions	1,3,5,6	All
**Must**	Date of Sample Collection	1,3,5,6	All
**Must**	Physical Location of Sample Collection	1,6	All
**Must**	Date of Sample Preparation	1	All
**Must**	Physical Location of Sample Preparation	1,6	All
**Must**	Sampling Protocol	1,5,6	All
**Must**	Sample ID (De-identified)	1,2,3,6	All
**Must**	Biospecimen Type	1,6	All
**Must If Applicable** ^a^	Clinical Diagnosis	1,6	All
**Must If Applicable** ^a^	Pathological State	1,6	All
**Should**	Sex assigned at Birth	1,3,7	Human
**Should**	Genetic Ancestry	1,3,5,6	Human
**Should**	Phenotypes	1,6	Human
**Should**	Anatomical Source of the Biospecimen	1,3,6	All
**Must**	Library Preparation	1,3,5,6	All
**Must If Applicable**	Targeted Coverage Regions (BED file)	2,6	All
**Should If Applicable**	Enrichment Kit Used	1,3,6	All
**Must**	Were Unique Molecular Identifiers Used?	1,3,5,6	All
**Must**	Sequencing Platform	1,3,6	All
**Must**	Sequencing Instrument Model	1,3,6	All
**Must**	Sequencing Method	1,3,6	All
**Must**	Sample Sequencing Date	1,5,6	All
**Must**	Sample Sequencing Physical Location	1,2,3,6	All
**Should**	Sequencer ID		All

Standards Legend: 1. FAIR Genomes 2. EDAM 3. MIxS GSC 4. Sequence Ontology 5. EFO 6. NCIT 7. GSSO.

a Metadata that must be provided when relevant to the specimen, study design, or analysis. If not applicable to the broader context, the field should be explicitly recorded as ‘not applicable’ or ‘not collected’.

### 2.2 Description of sequencing preparation and process

Structured metadata capturing sequencing library preparation and instrumentation support the explainability and readiness of genomic data for AI applications (Table 1). This includes capturing the library preparation and sequencing process (e.g. exome capture methods and PCR amplification), as well as the region coverage. If targeted panel or exome sequencing was performed, a browser extensible data (BED) file (Niu *et al*. 2022) should be provided that supplies the profiled regions or genomic locations to control for off-target or low sequence coverage results. Enrichment kits used for targeted sequencing experiments operate by different methods that should be described. Data producers should note what barcodes, unique molecular identifiers, and other synthetic sequences were used in this experiment, to inform the reanalysis and reuse of these data in downstream analysis pipelines. Metadata must also include information on the sequencing platform and instrument model, sequence pool and run identifiers, and the date and physical location each sample was sequenced. If applicable, samples sequenced across runs on multiple different machines should include these metadata for each applicable run contributing to the generated data.

### 2.3 Description of specifications for full genomic sequencing procedure

Sequencing data analysis pipelines are highly variable and essential to the interpretation of results generated from a sequencing experiment. Data analysis workflows often include bespoke data transformation and analysis methods that are definitional to the downstream variant call results. To control for some of these differences, we recommend including metadata specific to the processing and analysis of sequencing results (Table 2). Understanding the inherent purpose or intention behind an experiment is required to understand sequencing data. Data producers must include the analyses performed (e.g. phased genotyping, chromatin profiling, clonal variant calling) and the bioinformatics workflow used to produce the provided results files.

**Table 2:** ** **Metadata recommendations to describe genomic sequencing processing and procedure.

Requirement level	Name	Standards used	Sample from
**Must**	Analyses Performed	1,2	All
**Must**	Bioinformatics workflow and Software, including versioning, workflow definitions, algorithms, and any additional modifications	1,2,5,6	All
**Must**	Execution parameters and random seeds used for each analysis step	1,2,6	All
**Must**	Reference Genome or bundle used for alignment, including identifier, version, source pointer, and checksum or sequence digest	12,367	All
**Must**	Machine-actionable provenance record (e.g. W3C PROV or RO-Crate)	8,9	All
**Must**	Sequence Identifiers	6,7	All
**Should**	Specifications for Hardware	5,6	All
**Must**	Analysis Environment used, including container digest and dependency lockfile	6	All
Must	Date processed (every file)	Most have a general date	All
Must	Location/Institution processed (every file)	Most have a general location	All

Standards Legend: 1. FAIR Genomes 2. EDAM 3. MIxS GSC 4. Sequence Ontology 5. EFO 6. NCIT 7. GSSO 8. W3C PROV 9. RO-Crate.

Pipeline information should include the software used throughout the pipeline including the computational environment and version of the software (lockfile) with parameters used (including random seeds), and the specific algorithms (including deviations or modifications) used. Where possible, provenance should be captured using structured, machine-actionable workflow metadata rather than narrative descriptions. Existing provenance frameworks, such as W3C PROV and RO-Crate provide practice mechanisms for capturing this information and are discussed in greater detail in the Bridge2AI AI-readiness recommendations (Clark *et al*. 2024a). If the data has been aligned to a reference genome (e.g. GRCh38), the identity of the reference genome must be included, which should include at least the human-understandable alias (e.g. GRCh38), and also the GA4GH sequence collection digest, as well as a source accession if possible (e.g. GCF_000001405.40) (Campbell *et al*. 2025). Metadata about reference sequences should be stored alongside downstream alignments and variant calls for genomic data. Standard sequence digest approaches should be used to create identifiers for sequences, and digests should be stored in databases and file headers when used to produce file contents. We recommend that URL-safe sha512t24u (Wagner *et al*. 2021, Yates *et al*. 2021, Campbell *et al*. 2025) reference sequence digests be used for this purpose.

Specifications for hardware (e.g. GPU or FPGA) and the analysis environment used during the pipeline should be included to control for known/reported issues with these and the software being used for processing. Within the resulting data set, a descriptive file must be included that describes what each file in the dataset should contain (including format and descriptions), and the date and institution the file was processed. For example, if that dataset contains an annotated gVCF, this file should clearly explain what each column header means and the intended format and content of data in that column. We also recommend that consent and data use permissions be included to ensure data is not legally being misused; however, those data are ubiquitous to datasets and are outside the scope of these recommendations (Lawson *et al*. 2021).

### 2.4 Capturing adequate quality control data

Data that has been aligned should include base call and read mapping quality, paired read data (as applicable) and depth of coverage scores (Table 3). The ideal depth of coverage scores are calculated post-deduplication to accurately reflect the usable reads for variant calling. Scores should include mean coverage, and if the experiment is a targeted capture, the quality control data should also capture the percent of target bases with suitable coverage (e.g. 30× depth), as this is a key metric for assessing the quality of the capture kit and sequencing run. Other quality metrics that should be provided include (1) error rate summary, (2) sequenced GC content, (3) breadth of coverage (for alignment data), and (4) chromosomal ploidy, if known (including sex chromosomes). Depending on the sequencing assay and analysis workflow, additional quality control metrics (e.g. duplication rate, mapping rate, contamination estimates, library complexity, or sample identity concordance) can provide valuable context for downstream interpretation and AI applications.

**Table 3:** ** **Recommendations for quality control metrics.

Requirement level	Name	Example value	Sample from
**Must**	Read Mapping Quality^a^	10	All
**Must**	Depth of Coverage Scores^b^	45	All
**Should If Applicable**	Mean Coverage^c^	100	All
**Should If Applicable**	Percent of Target Bases with Suitable Coverage	75%	All
**Should**	Error Summary Metrics	‘.error_by_base_quality’ file	All
**Should**	GC Content	25%	All
**Should**	Breadth of Coverage (alignment data)	95%	All
**Should If Applicable**	Duplication Rate	12%	All
**Should If Applicable**	Mapping Rate	98%	All
**Should If Applicable**	Contamination Estimate	0.5%	All
**Should If Applicable**	Library Complexity	2.1 × 10^8^ unique molecules	All
**Should If Applicable**	Sample Identity Concordance	Concordant with matched sample	Human
**Should**	Genotypic Sex	XX Genotype	Human

a The ideal metric is per-read alignment.

b The coverage score at a specific sequence location of interest.

c The mean value over all sequenced regions.

### 2.5 Data storage formats

Appropriate file formats to store genome sequences include FASTQ (reads only), BAM (reads with alignments), and CRAM (compressed reads with alignments). Read data only needs to be preserved in one of these formats. While BAM and CRAM files allow for storing alignments without accompanying read sequence or base quality scores, we recommend that sequence read and alignment data always be stored together in CRAM files (Supplementary Table 2, available at supplementary material  *Bioinformatics Advances* online).

Variant call data should include variant quality scores, the total number of reads covering the variant, the number of reads supporting a reference allele call, and the number of reads supporting an alternate allele call (Table 4). As highlighted in the NHGRI FAIR Data Workshop, for semantically precise representation of variant data for use by AI systems, we recommend the use of data standards compatible with the GA4GH Variation Representation Specification (VRS) (Wagner *et al*. 2021). The VRS standard provides a semantically-rich schema that works as a *lingua franca* for representing all forms of genomic variation, an information model designed for cross-resource genomic data integration, and a standard method for generating globally unique computational identifiers. VRS complements existing variant file formats by providing standardized representations and identifiers for genomic variants while preserving sample-specific sequencing evidence.

**Table 4:** ** **Metadata recommendations to support variant call data.

Requirement level	Name	Example value	Sample from
**Must**	Variant Quality Score	20	All
**Must**	Filter	PASS/FAIL	All
**Must**	Total Reads Covering the Variant	150	All
**Must**	Number of Reads Supporting a Reference Allele Call	75	All
**Must**	Number of Reads Supporting an Alternate Allele Call	75	All
**Must**	VRS Variant Object Data	<VRS Location/VRS State>	All
**Should**	VRS Identifiers	ga4gh: VA.EgHPXXhULTwoP4-ACfs-YCXaeUQJBjH_	All
**May**	Genotype Quality Score	89	All

For storing small variant (e.g. SNVs and indels) data from large-scale sequencing studies, we recommend these data are stored as gVCF files, based on the GA4GH Variant Call Format (VCF) (at least version 4.2), with version 4.3 preferred. Because gVCF representations may vary across variant callers, the corresponding variant calling software and version should be recorded as well. These files should retain sample-level information (including genotypes, zygosity, quality metrics, filtering information, phasing, and supporting read evidence) while VRS objects provide interoperable representations of the underlying variants. Users may also include other downstream formats, such as GFF3 to represent annotated genomic features. For pipelines using the GA4GH VCF file format, these files can be readily made VRS-compatible using the VRS-Python software stack.

### 2.6 Autism genotype-phenotype associations as an example of the importance of metadata capture

Proper capture and documentation of metadata is essential for enabling genomics and precision medicine through AI operations. Without the proper context and procedural details, an AI model trained using ill-described data runs the risk of generating insights linked to experimental artifacts rather than true biological effects. To illustrate this, we have provided a sample use case in the observation of Autism genotype-phenotype associations (Supplementary Data, available at supplementary material  *Bioinformatics Advances* online). In brief, we demonstrate how the absence of key metadata from genome sequencing samples used to train an AI model resulted in preliminary findings that were more closely associated with sample source (saliva vs. blood) than any real biological associations. By demonstrating these risks, we hope to further persuade the proper capture and standardization of metadata generated from genomics approaches to enable robust AI applications.

## 3 Conclusion

The emergence of widespread AI technologies has created opportunities for the application of genomics data in scalable, machine-driven biomedical discovery. Alongside this potential, however, also comes a new responsibility to ensure that genomics datasets are AI-ready; that is, data must be explainable, reusable, and computationally available for precise usage. Unlike conventional data reuse, AI-ready datasets must preserve sufficient structured metadata to enable automated and reproducible interpretation. The recommendations and standards for metadata discussed in this report represent reliable consensus guidelines that, when followed, will improve the AI-ready status of genomic sequencing datasets. To support practical implementation of these recommendations, we have developed a companion GitHub repository containing implementation guidance, reusable metadata templates, and links to relevant community standards (https://github.com/bridge2ai/genomic-information-standards). This repository is intended to serve as a living community resource that will evolve alongside emerging standards and best practices.

The primary limitation of these recommendations are their limited scope, which is focused specifically on small variant detection, and therefore does not fully capture all the variability present in genomics research. Nevertheless, many of the recommendations regarding metadata capture, provenance, quality control, and standardized identifiers are broadly applicable across emerging sequencing technologies with additional, technology-specific standards likely needed as these fields continue to evolve. We encourage readers to follow the guidance of the GA4GH for recommendations specific to additional genomics technologies when preparing AI-ready genomics data.

## Supplementary Material

vbag305_Supplementary_Data

## Data Availability

All data discussed in this study are provided in the article and its supplementary data files. The complimentary implementation guidance GitHub repository is available from https://github.com/bridge2ai/genomic-information-standards. No additional datasets were generated beyond those included in the manuscript and supplementary materials.
