# Supplementary material for: Bridge2AI recommendations for AI-ready genomic data
Source: Bioinform Adv. 2026 Oct 25;6(1):vbag305. doi: 10.1093/bioadv/vbag305 (PMC13695847; doi:10.1093/bioadv/vbag305)
Supplement: Supplementary file 1 [file vbag305_supplementary_data.zip › GIST - Supplementary Autism Use Case - Final.docx]

## ***Use Case: The Essential Role of Metadata in Refining Genotype-Phenotype Associations for Autism Sequencing Projects***

### **Objective**

To show the critical value of metadata in discovering the genotype-phenotype associations accurately, illustrated in the context of a large-scale autism sequencing project undertaken by genome centers B1 and B2.

**Nature of this use case**

This is an illustrative composite grounded in a real large-scale autism genome sequencing effort. The scenario has been reconstructed and in part synthesized, and site identities and site-level details generalized, to prevent re-identification and comply with data use restrictions. It is not a report of measured outcomes: the metadata availability in Table 1 reflects the underlying effort, but model performance and any quantities are illustrative of the expected effect, not empirical results.

### **Background**

The project embarked on genome sequencing (GS) to investigate the genetic intricacies of autism. Despite advanced sequencing and analytical methods, initial analyses by B1 and B2 faced challenges in identifying genotype-phenotype associations accurately.

### **Initial Approach and Challenges**

●	Due to the quality of the collected sample and concern of contamination during sample collection, GS conducted in both centers primarily used DNA extracted from blood, with a subgroup using DNA from saliva samples. However, the proportion of saliva samples was different between B1 and B2

●	Each center initiated GS with slight variations in PCR chemistry: B1 utilized 2-channel while B2 employed a mix of 2-channel and 4-channel chemistry sequencers.

●	Different software pipelines were used for alignment and variant calling across centers, introducing variability in data processing: BWA-MEM and GATK at B1, and DRAGMAP and DRAGEN at B2.

●	The phenotypic measures comprised 27 variables, both categorical and continuous, for neuro-cognitive development.

An initial AI model was developed, pre-trained on population-scale genomic variants and phenotypes, and then fine-tuned with autism GS data from both centers. However, the sample-source metadata (saliva vs. blood) not being machine-actionable, along with subtle phenotypic score differences caused by discrepancies in sequencing and data processing, resulted in preliminary findings that were linked to technical artifacts rather than to genuine biological associations.

### **Metadata Integration**

Recognizing the need to overcome these challenges, a second AI model was conceptualized with a comprehensive metadata approach:

1. **Pre-training** involved both sample-level and variant-level metadata, including details about the sequencing chemistry and procedures unique to centers B1 and B2.
2. **Fine-tuning** was enhanced with GS metadata, encompassing sequencing depth, alignment, and variant calling nuances, alongside the 27 neuro-cognitive development measures.

### **Outcomes**

With sample-source and processing metadata available, the second model can separate center- and chemistry-driven variation from biological signal—variation the first model cannot distinguish. Associations that would otherwise be misconstrued as biologically relevant, when they are in fact artifacts of variation in sequencing methods and sample sources, can be filtered out. The refined model would be expected to recover associations that the first model’s artifacts obscure, where the first model’s apparent associations trace predominantly to the kind of artifact expected here—indel-rich regions, homopolymers, and center-specific SNVs.

### **Impact**

●	**Precision in Discovery:** Integrating detailed machine-actionable metadata directly addresses the initial model’s core limitation—its inability to separate technical variation from biological signal—which is what allows authentic associations to be distinguished from artifacts.

●	**Insights into Methodological Variability:** The case illustrates how methodological differences between sequencing centers can propagate into apparent research outcomes, arguing for a standardized approach to data collection and analysis.

●	**Guidance for Future Genomic Research:** .It illustrates the critical role of machine-actionable metadata in genomic studies, offering a template for future projects to guard against artifact-driven associations.

### **Conclusion**

The autism sequencing scenario, from artifact-driven initial associations to their separation once sample-source and processing context is available, illustrates the value of machine-actionable metadata in genomic research. This use case argues for the meticulous inclusion of such metadata and offers a template for future work aiming to make genomic studies as precise and informative as possible.

As this use case illustrates, enabling precision medicine through AI operations requires that genomic data generators capture key metadata and experimental context in machine-actionable form. Doing so allows covariates to be controlled and makes it possible to separate real associations from artifacts during AI operations.

Table 1. Metadata

| Metadata field | Status in underlying effort |
| --- | --- |
| Sample source (blood / saliva) | Present, not machine-actionable |
| Saliva:blood proportion per center | Derivable from sample source (not precomputed) |
| Sequencing chemistry (2-vs-4 channel) | Present, not machine-actionable |
| Alignment pipeline (BWA-MEM / DRAGMAP) | Recorded, not standardized |
| Variant-calling pipeline (GATK / DRAGEN) | Recorded, not standardized |
| Sequencing depth | Recorded |
| Phenotype (27 measures) | Recorded |
| Site identifier | Recorded (generalized here) |
