# Supplementary material for: Bridge2AI recommendations for AI-ready genomic data
Source: Bioinform Adv. 2026 Oct 25;6(1):vbag305. doi: 10.1093/bioadv/vbag305 (PMC13695847; doi:10.1093/bioadv/vbag305)
Supplement: Supplementary file 1 [file vbag305_supplementary_data.zip › GIST - Supplementary Tables - Final.docx]

| **Term** | **Ontology/Reference** |
| --- | --- |
| sampling protocol | <https://www.ebi.ac.uk/ols4/ontologies/efo/classes?short_form=EFO_0005518> |
| biospecimen type | <https://ontobee.org/ontology/NCIT?iri=http://purl.obolibrary.org/obo/NCIT_C70713> |
| key clinical diagnosis | <https://ontobee.org/ontology/NCIT?iri=http://purl.obolibrary.org/obo/NCIT_C15607> |
| pathological state | <https://github.com/fairgenomes/fairgenomes-semantic-model/blob/main/lookups/PathologicalState.txt> |
| sex assigned at birth | <https://ontobee.org/ontology/GSSO?iri=http://purl.obolibrary.org/obo/GSSO_009418> |
| genetic ancestry | <https://ontobee.org/ontology/NCIT?iri=http://purl.obolibrary.org/obo/NCIT_C176763> |
| phenotypes | <https://ontobee.org/ontology/NCIT?iri=http://purl.obolibrary.org/obo/NCIT_C16977> |
| library preparation | <https://github.com/fairgenomes/fairgenomes-semantic-model/blob/main/lookups/NGSKits.txt> |
| unique molecular identifiers | <https://www.ebi.ac.uk/ols4/ontologies/efo/classes?short_form=EFO_0010199> |
| sequencing platform | <https://github.com/fairgenomes/fairgenomes-semantic-model/blob/main/lookups/SequencingPlatform.txt> |
| sequencing instrument model | <https://github.com/fairgenomes/fairgenomes-semantic-model/blob/main/lookups/SequencingInstrumentModels.txt> |
| sequencing method | <https://github.com/fairgenomes/fairgenomes-semantic-model/blob/main/lookups/SequencingMethods.txt> |
| analyses performed | <https://github.com/fairgenomes/fairgenomes-semantic-model/blob/main/lookups/AnalysesPerformed.txt> |
| reference genome | <https://github.com/fairgenomes/fairgenomes-semantic-model/blob/main/lookups/GenomeAccessions.txt> |
| genotypic sex | <https://github.com/fairgenomes/fairgenomes-semantic-model/blob/main/lookups/GenotypicSex.txt> |
| FASTQ format | <https://maq.sourceforge.net/fastq.shtml> |
| BAM format | <https://samtools.github.io/hts-specs/SAMv1.pdf> |
| CRAM format | <https://samtools.github.io/hts-specs/CRAMv3.pdf> |

**Supplementary Table 1. Supporting definitions and ontology references for genomics metadata terminology.** All ontology and online reference resources were accessed July 2026.

| Requirement Level | Data Storage Formats | Contains | Sample Derived From |
| --- | --- | --- | --- |
| Must | CRAM/BAM/SAM | Alignment data | All |
| Must | gVCF (min version 4.2) | Genomic variant calls | All |
| Must | FASTA | Nucleotide sequences | All |
| Must | FASTQ | Reads with quality scores | All |
| Must | GFF | Genomic feature annotations | All |

**Supplementary Table 2. Data storage formats**
